# Smad7 protects against chronic aristolochic acid nephropathy in mice

**DOI:** 10.18632/oncotarget.3718

**Published:** 2015-03-30

**Authors:** Xiao-Yu Dai, Li Zhou, Xiao-Ru Huang, Ping Fu, Hui-Yao Lan

**Affiliations:** ^1^ Department of Medicine and Therapeutics, and Li Ka Shing Institute of Health Sciences, and Shenzhen Research Institute, The Chinese University of Hong Kong, Hong Kong, China; ^2^ Division of Nephrology, West China Hospital of Sichuan University, Chengdu, China

**Keywords:** chronic aristolochic acid nephropathy, Smad7, renal fibrosis, renal inflammation

## Abstract

Chronic Aristolochic Acid Nephropathy (AAN) is a progressive chronic kidney disease related to herb medicine. However, treatment for chronic AAN remains ineffective. We report here that Smad7 is protective and has therapeutic potential for chronic AAN. In a mouse model of chronic AAN, progressive renal injury was associated with a loss of renal Smad7 and disruption of Smad7 largely aggravated the severity of chronic AAN as demonstrated by a significant increase in levels of 24-hour urinary protein excretion, serum creatinine, and progressive renal ﬁbrosis and inflammation. In contrast, restored Smad7 locally in the kidneys of Smad7 knockout mice prevented the progression of chronic AAN. Further studies revealed that worsen chronic AAN in Smad7 knockout mice was associated with enhanced activation of TGF-β/Smad3 and NF-κB signaling pathways, which was reversed when renal Smad7 was restored. Importantly, we also found that overexpression of Smad7 locally in the kidneys with established chronic AAN was capable of attenuating progressive chronic AAN by inactivating TGF-β/Smad3-medated renal fibrosis and NF-κB-driven renal inflammation. In conclusion, Smad7 plays a protective role in the pathogenesis of chronic AAN and overexpression of Smad7 may represent a novel therapeutic potential for chronic AAN.

## INTRODUCTION

Aristolochic acid nephropathy (AAN) is initially called Chinese-herb nephropathy before aristolochic acid (AA) is known as a cause of AAN [1, 2]. AAN is first reported in Belgium in patients with prolonged intake of Chinese herbs [3]. Since then, new cases of AAN are regularly reported because of the use of herbal therapy worldwide [4-6]. Clinically, patients with AAN exhibit a rapidly progressive interstitial nephritis leading to end-stage renal disease [3, 7, 8]. A similar clinical course is also observed in experimental animals treated with AA [9]. Pathologically, chronic AAN is characterized by extensive tubulointerstitial ﬁbrosis with atrophy and loss of the tubules in both patients and animal models of AAN [7-9]. However, the mechanisms of AAN remain largely unclear and no treatment for chronic AAN is yet available.

In the context of renal fibrosis, TGF-β1 acts by stimulating Smad3 to mediate fibrosis, which is negatively regulated by Smad7 [10, 11]. In a mouse model of chronic AAN, deletion of Smad3 gene protects against progressive renal fibrosis, revealing a pathogenic role for Smad3 in the development of chronic AAN [12]. In addition, Smad7 is also known to be a negative regulator of NF-κB signaling [13]. Deletion of Smad7 enhances renal ﬁbrosis and inﬂammation, whereas, overexpression of Smad7 attenuates renal ﬁbrosis and inﬂammation by blocking the activation of both TGF-β/Smad and nuclear factor-κB (NF-κB) signaling pathway in a variety of animal models with kidney diseases [14-22]. However, role of Smad7 in chronic AAN remains unexplored. Thus, in the current study, we investigated the mechanisms and potential role of Smad7 in a mouse model of chronic AAN induced in Smad7 gene knockout (KO) mice and by restoring Smad7 locally in the kidneys of Smad7 KO mice. Furthermore, we also showed that overexpression of Smad7 in the established chronic AAN was capable of attenuating the progression of chronic AAN.

## RESULTS

### Deletion of Smad7 results in more severe kidney injury in a mouse model of chronic AAN

**Figure 1:**
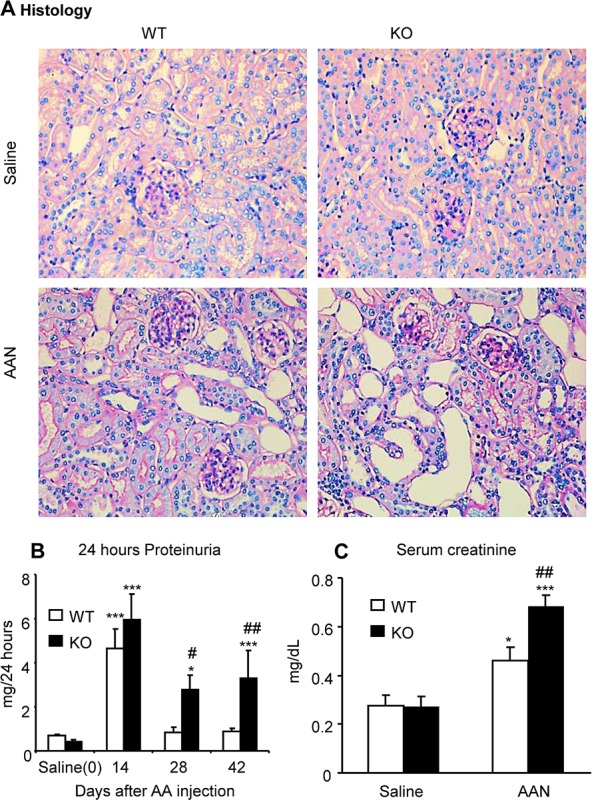
Deletion of Smad7 promotes chronic AAN **A**: Histology (PAS-stained sections) at day 42 after induction of AAN. **B**: Proteinuria (24-h) over the 42-day experimental time course. **C**: Serum creatinine at day 42 after induction of AAN. Note that disruption of Smad7 enhances AA-induced renal injury, including severe histological damage such as dilated and bared tubular basement membrane, higher levels of proteinuria and serum creatinine when compared with Smad7 WT mice. Data are expressed as mean ±SE for groups of 6 mice. *P < 0.05, ***P < 0.001 compared with saline control mice. ^#^*P* < 0.05, ^##^*P* < 0.01 compared with Smad7 WT mice with chronic AAN mice. Magnifications: x200.

**Figure 2:**
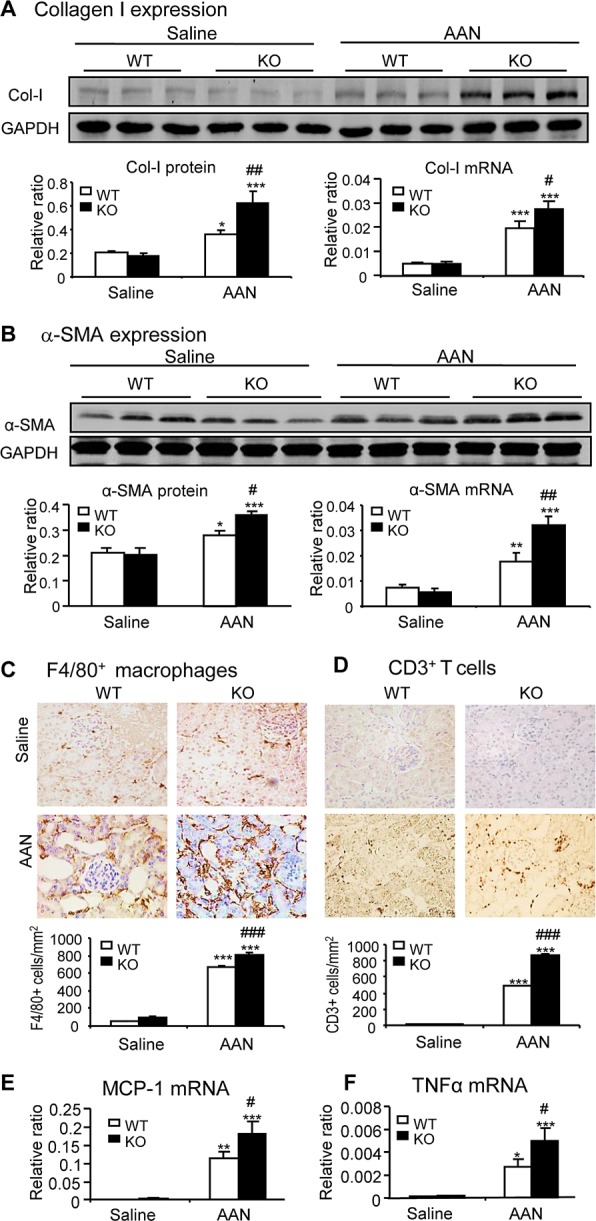
Disruption of Smad7 enhances AA-induced renal fibrosis and inflammation at day 42 after induction of AAN **A** and **B**: Renal collagen I and α-SMA mRNA and protein expression by real-time PCR and western blot analysis. **C** and **D**: Renal infiltration of F4/80^+^macrophages and CD3^+^ T cells detected by immunohistochemistry. E and F: MCP-1 and TNFα mRNA expression detected by real-time PCR. Results show that compared with the WT mice, Smad7 KO mice show a significant increase in renal fibrosis and inflammation. Data are expressed as mean ± SE for groups of 6 mice. *P < 0.05, **P < 0.01, ***P < 0.001 compared with the saline control mice. ^#^*P* < 0.05, ^##^*P* < 0.01, ^###^*P* < 0.001 compared with Smad7 WT mice with chronic AAN mice. Magnification: x400.

Compared with normal mice, after intraperitoneal injection of AA for 6 weeks, Smad7 WT mice developed chronic AAN as evidenced by severe tubulointerstitial ﬁbrosis accompanied by dilated tubular lumens with bared tubular basement membrane and an increase in 24h proteinuria and serum creatinine (Fig. 1). All these changes were further acerbated in Smad7 KO mice with chronic AAN (Fig. 1). Analysis with immunohistochemistry, western blot, and real-time PCR also revealed that compared to WT mice, deletion of Smad7 largely enhanced expression of collagen I and α-SMA and promoted renal inflammation including expression of tumor necrosis factor-α (TNFα), chemokine monocyte chemoattractant protein-1 (MCP-1), and a marked infiltration of F4/80+ macrophages and CD3+ T cells (Fig. 2 and Fig. S1 and S2).

### Enhanced activation of TGF-β/Smad3 and NF-κB signaling is a key mechanism by which deletion of Smad7 promotes chronic AAN injury

**Figure 3:**
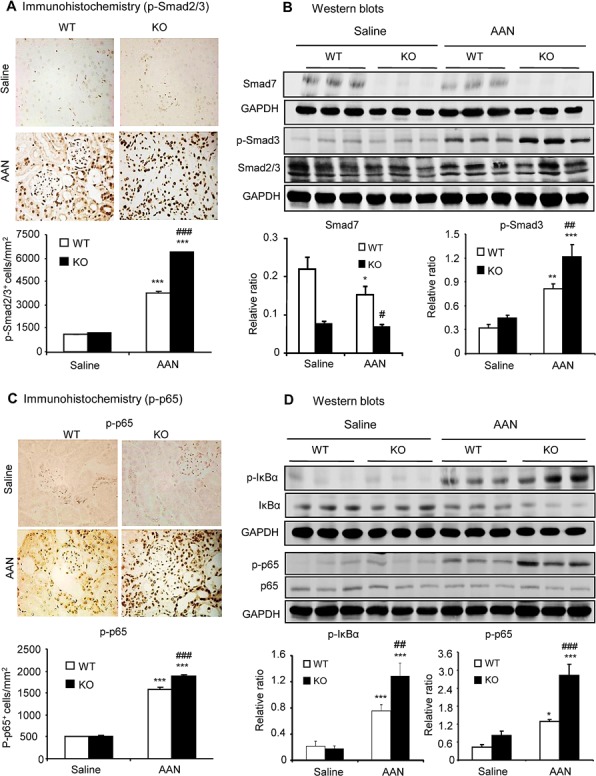
Disruption of Smad7 sustains TGF-β/Smad and NF-κB signaling in the kidney with chronic AAN at day 42 after induction of AAN **A**: Phosphorylated Smad2/3 nuclear translocation by immunohistochemistry. **B**: Smad7 expression and phosphorylated Smad3 (p-Smad3) by western blotting. **C**: Phosphorylated NF-κB/p65 nuclear translocation by immunohistochemistry. **D**: Phosphorylation of IκBα and NF-κB/p65 by western blotting. Note that compared to normal Smad7 WT mice, Smad7 is significantly reduced with a marked activation of both TGF-β/Smad3 and NF-κB signaling in chronic AAN, which becomes maximal in the AAN kidney of Smad7 KO mice. Data are expressed as mean ± SE for groups of 6 mice. *P < 0.05, **P < 0.01, ***P < 0.001 compared with saline control mice. ^#^*P* < 0.05, ^##^*P* < 0.01, ^###^*P* < 0.001 compared with Smad7 WT mice with chronic AAN mice. Magnification: ×400.

To investigate the mechanisms by which deletion of Smad7 promoted renal fibrosis and inflammation in mice with chronic AAN, we examined TGF-β/Smad and NF-κB signaling pathways since both pathways are critical in the development of renal fibrosis and inflammation in many pathological conditions [10]. Immunohistochemistry, real-time PCR and western blot analysis revealed that chronic AA injection significantly up-regulated renal TGF-β1 with over-activation of TGF-β/Smad3 signaling, which was associated with a significant reduction in renal Smad7 protein in Smad7 WT mice (Fig. 3A and 3B and Fig. S3). Deletion of Smad7 sustained further activation of TGF-β/Smad3 signaling (Fig. 3A and 3B and Fig. S3). Furthermore, disruption of Smad7 also enhanced activation of NF-κB signaling as demonstrated by higher levels of NF-κB/p65 phosphorylation and phosphorylated p65 nuclear translocation, which was associated with a marked reduction in the NF-κB inhibitor, IκBα, on the basis of increased IκBα degradation by phosphorylation (Fig. 3C and 3D).

### Restored renal Smad7 rescues AA-induced renal dysfunction, fibrosis and inflammation on Smad7 KO mice

**Figure 4:**
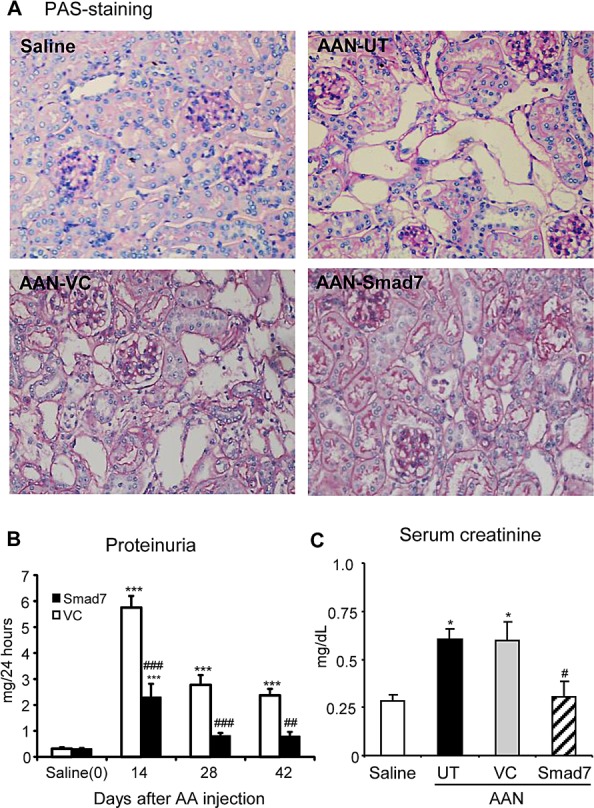
Restored renal Smad7 rescues the development of chronic AAN in Smad7 KO mice **A**: Histology (PAS-stained sections) at day 42 after induction of AAN. **B**: Proteinuria (24-h) over the 42-day period. **C**: Serum creatinine at day 42 after induction of AAN. Note that restored renal Smad7 on Smad7 KO mice with AAN (AAN-Smad7) inhibits AA-induced progressive renal injury, including severe histological damage such as dilated and bared tubular basement membrane, higher levels of proteinuria and serum creatinine when compared with control Smad7 KO mice with chronic AAN without treatment (AAN-UT) or treated with vector control (AAN-VC). Data are expressed as mean ± SE for groups of 6 mice. *P < 0.05, *P < 0.01, ***P < 0.001 compared with saline control mice. ^#^*P* < 0.05, ^##^*P* < 0.01, ^###^*P* < 0.001 compared with Smad7 KO mice with chronic AAN treated with or without VC. Magnifications: x200.

**Figure 5:**
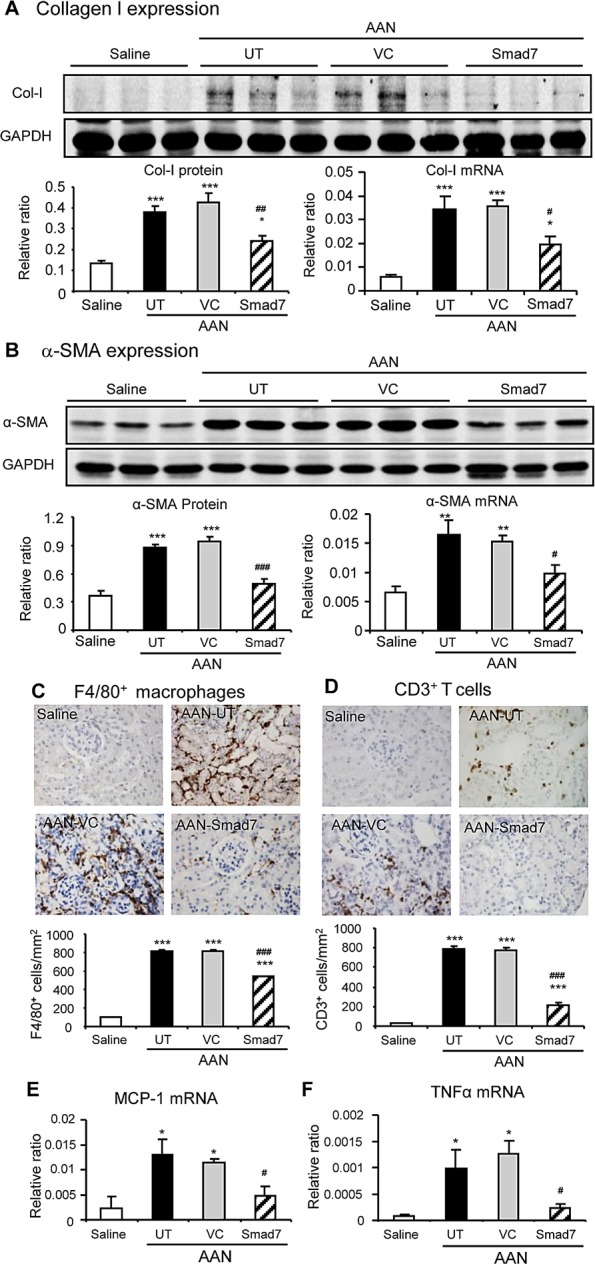
Restored renal Smad7 inhibits AA-induced renal fibrosis and inflammation in Smad7 KO mice at day 42 after induction of AAN **A** and **B**: Renal collagen I and α-SMA mRNA and protein expression by real-time PCR and western blot analysis. **C** and **D**: Renal infiltration of F4/80^+^macrophages and CD3^+^ T cells detected by immunohistochemistry. **E** and **F**: MCP-1 and TNFα mRNA expression detected by real-time PCR. Results show that compared to Smad7 KO mice with chronic AAN without treatment (AAN-UT) or treated with vector control (AAN-VC), restored renal Smad7 on Smad7 KO with AAN (AAN-Smad7) largely blocks renal fibrosis and inflammation. Data are expressed as mean ± SE for groups of 6 mice. *P < 0.05, **P < 0.01, ***P < 0.001 compared with the saline control mice. ^#^*P* < 0.05, ^##^*P* < 0.01, ^###^*P* < 0.001 compared with Smad7 KO mice with chronic AAN treated with or without VC. Magnification: x400.

**Figure 6:**
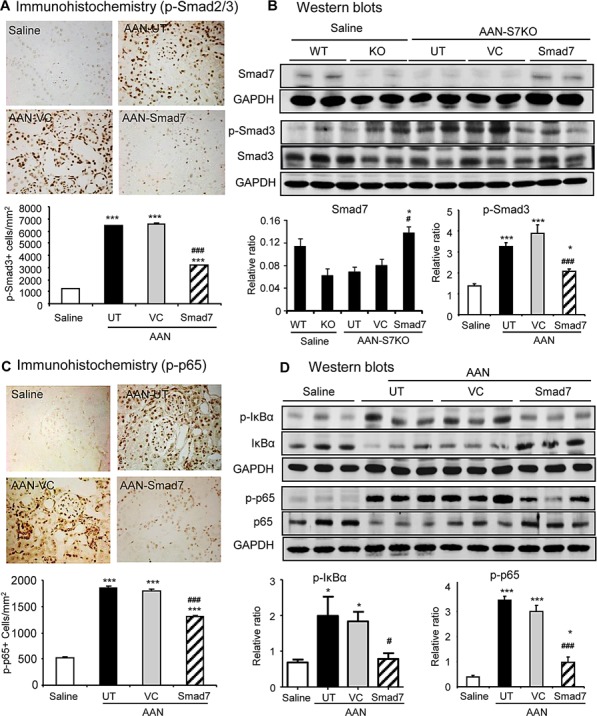
Restored renal Smad7 blocks TGF-β/Smad and NF-κB signaling in the kidney of Smad7 KO mice with chronic AAN at day 42 after induction of AAN **A**: Phosphorylated Smad2/3 nuclear translocation by immunohistochemistry. **B**: Smad7 expression and phosphorylated Smad3 (P-Smad3) by western blotting. **C**: Phosphorylated NF-κB/p65 nuclear translocation by immunohistochemistry. **D**: Phosphorylation of IκBα and NF-κB/p65 by western blotting. Note that compared to Smad7 KO mice with chronic AAN without treatment (AAN-UT) or treated with vector control (AAN-VC), restored renal Smad7 in the AAN kidney of Smad7 KO mice (AAN-Smad7) largely blocks a marked activation of both TGF-β/Smad3 and NF-κB signaling. Data are expressed as mean ± SE for groups of 6 mice. *P < 0.05, **P < 0.01, ***P < 0.001 compared with saline control mice. ^#^*P* < 0.05, ^##^*P* < 0.01, ^###^*P* < 0.001 compared with Smad7 KO mice with chronic AAN treated with or without VC. Magnification: x400.

To further confirm the protective role of Smad7 in chronic AAN, we locally delivered an inducible Smad7 gene into the kidneys of Smad7 KO mice via tail vein using an ultrasound-microbubble-mediated technique as previously described [14-17]. We found that restored renal Smad7 largely prevented AA-induced chronic AAN such as dilated and bared tubular basement membrane changes in tubulointerstitium and attenuated 24h proteinuria and serum creatinine when compared with those treated with or without empty vector control (Fig. 4). Immunohistochemistry, western blot, and real-time PCR also showed that restored renal Smad7 blocked AA-induced severe renal fibrosis such as upregulation of collagen I and α-SMA and inhibited renal inflammation including F4/80+ macrophages and CD3+ T cells and upregulation of MCP-1 and TNFα (Fig. 5 and Figs. S4 and S5). Western blot analysis clearly revealed that the inhibitory effect of Smad7 on renal fibrosis and inflammation was associated with the restoration of exogenous Smad7, thereby inhibiting TGF-β/Smad signaling by suppressing the phosphorylation of Smad3 and upregulation of TGF-β1 (Fig. 6A and 6B and Fig.S6) and attenuating NF-κB signaling by lowering phosphorylated IκBα and NF-κB/p65 in the AAN kidney (Fig. 6C and 6D).

### Smad7 has therapeutic potential for chronic AAN

**Figure 7:**
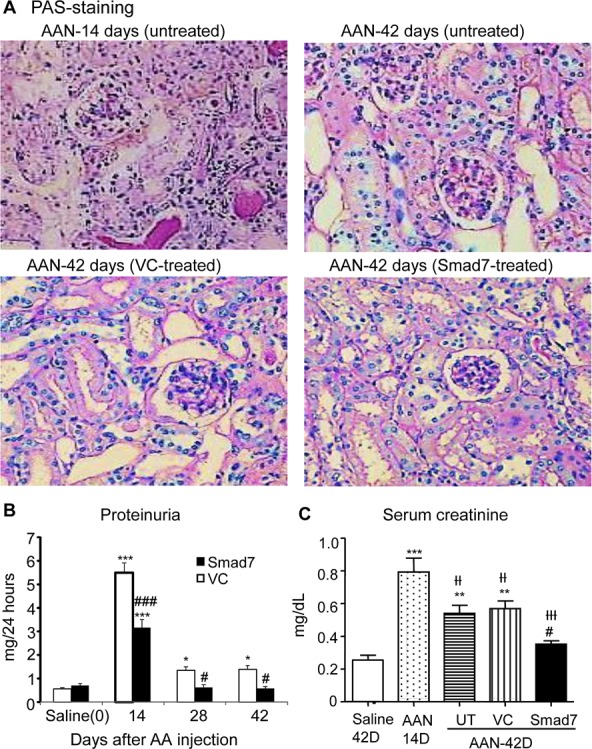
Smad7 treatment locally in the kidney with established chronic AAN attenuates progressive renal injury in Smad7 WT mice **A**: Histology (PAS-stained sections) at day 42 chronic AAN after the 28-day Smad7 treatment (days14-42). **B**: Proteinuria (24-h) over the 28-day Smad7 treatment (days 14-42). **C**: Serum creatinine at day 42 chronic AAN after the 28-day Smad7 treatment (days 14-42). Note that ultrasound-microbubble-mediated Smad7 overexpression (Smad7) in the kidney with the established AAN (AAN-Smad7) from day 14 to day 42 after induction of AAN attenuates progressive renal injury, including severe histological damage such as dilated and bared tubular basement membrane at day 42, higher levels of serum creatinine at day 42, and proteinuria over the 28-day treatment period (days 14-42) when compared with chronic AAN kidney without treatment (AAN-UT) or treated with vector control (AAN-VC). Data are expressed as mean ± SE for groups of 6 mice. *P < 0.05, *P < 0.01, ***P < 0.001 compared with saline control mice. ^#^*P* < 0.05, ^##^*P* < 0.01, ^###^*P* < 0.001 compared with Smad7 WT mice with chronic AAN treated with or without VC. ^††^*P* < 0.01, ^†††^*P* < 0.001 compared with day 14 disease before Smad7 treatment. Magnifications: x200.

**Figure 8:**
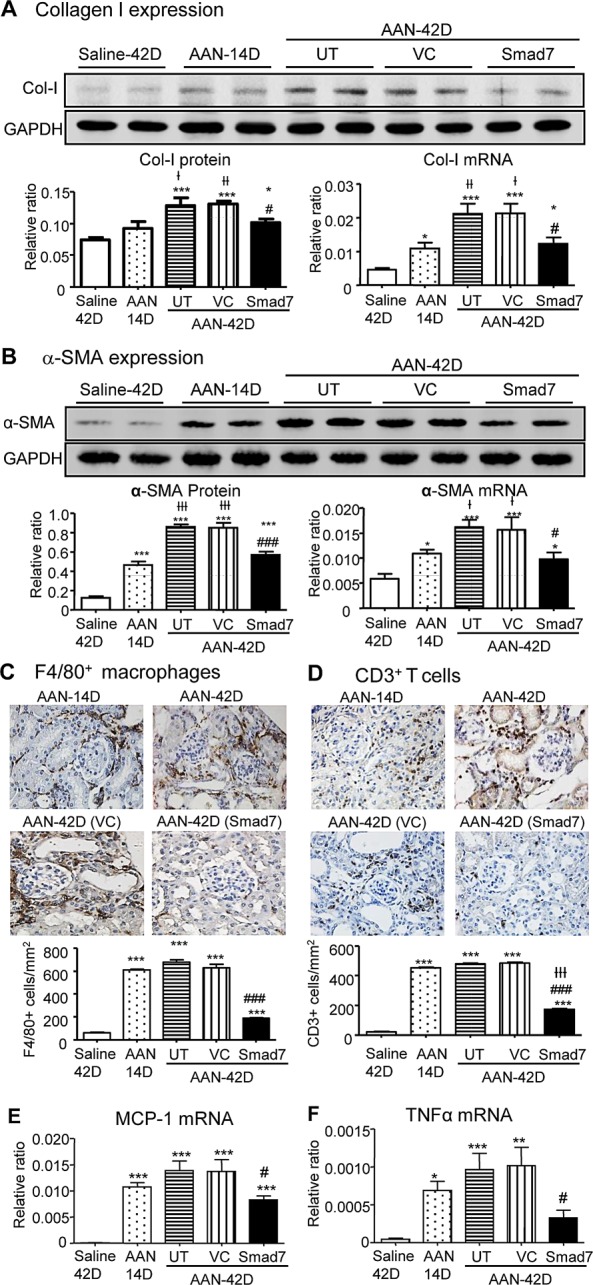
Local Smad7 therapy inhibits progressive renal fibrosis and inflammation in Smad7 WT mice with established chronic AAN at day 42 **A** and **B**: Renal collagen I and α-SMA mRNA and protein expression by real-time PCR and western blot analysis. **C** and **D**: Renal infiltration of F4/80^+^ macrophages and CD3^+^ T cells detected by immunohistochemistry. **E** and **F**: MCP-1 and TNFα mRNA expression detected by real-time PCR. Results show that compared to Smad7 WT mice with chronic AAN without treatment (AAN-UT) or treated with vector control (AAN-VC), Smad7 treatment (AAN-Smad7) locally in the kidney with the established AAN from day 14 to day 42 blocks renal fibrosis and inflammation at day 42. Data are expressed as mean ± SE for groups of 6 mice. *P < 0.05, **P < 0.01, ***P<0.001 compared with saline control mice. ^#^*P* < 0.05, ^##^*P* < 0.01, ^###^*P* < 0.001 compared with Smad7 WT mice with chronic AAN treated with or without VC. ^†^*P* < 0.05, ^††^*P* < 0.01, ^†††^*P* < 0.001 compared with AAN at day 14 before Smad7 treatment. Magnification: x400.

To explore whether Smad7 has therapeutic effect on chronic AAN, we transferred Smad7 gene into the diseased kidney of Smad7 WT mice with established chronic AAN at day 14 after AA administration. Results showed that compared with control-treated AAN mice at day 42, AAN mice received Smad7 treatment from day 14 to day 42 after induction of AAN were protected from progressive renal histological and functional injury (Fig. 7). Further studies also detected that Smad7 treatment largely inhibited a marked upregulation of collagen I and α-SMA and suppressed renal inflammation including expression of MCP-1 and TNFα, and infiltration of macrophages and T cells within the diseased kidney (Fig. 8 and Figs. S7 and S8).

**Figure 9:**
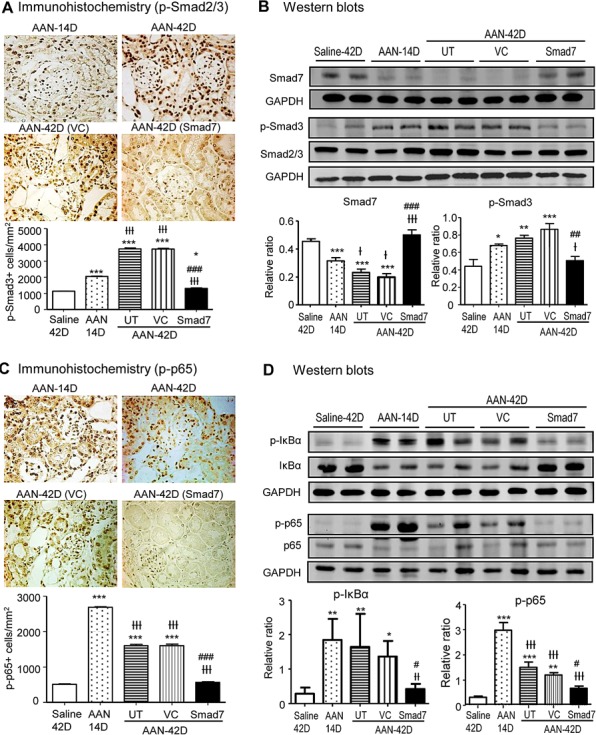
Smad7 treatment locally in the kidney with the established chronic AAN blocks activation of TGF-β/Smad and NF-κB signaling **A**: Phosphorylated Smad2/3 nuclear translocation by immunohistochemistry. **B**: Smad7 expression and phosphorylated Smad3 (p-Smad3) by western blotting. **C**: Phosphorylated NF-κB/p65 nuclear translocation by immunohistochemistry. **D**: Phosphorylation of IκBα and NF-κB/p65 by western blotting. Note that compared to Smad7 WT mice with chronic AAN without treatment (AAN-UT) or treated with vector control (AAN-VC), Smad7 treatment (AAN-Smad7) locally in the kidney with the established AAN from day 14 to day 42 largely blocks a marked activation of both TGF-β/Smad3 and NF-κB signaling in the AAN kidney at day 42. Data are expressed as mean ± SE for groups of 6 mice. *P < 0.05, **P < 0.01, ***P < 0.001 compared with saline control mice. ^#^*P*<0.05, ^##^*P* < 0.01, ^###^*P* < 0.001 compared with Smad7 WT mice with chronic AAN at day 42 treated with or without VC. ^†^*P* < 0.05, ^††^*P* < 0.01, ^†††^*P* < 0.001 compared with day 14 disease before Smad7 treatment. Magnification: x400.

Next, we examined the therapeutic mechanisms by which Smad7 treatment attenuates AA-induced renal inflammation and fibrosis. As shown in Fig. 9 and Fig. S9, immunohistochemistry, real-time PCR, and western blot analysis revealed that treatment with Smad7 in the established chronic AAN over days 14-42 abolished AA-induced activation of TGF-β/Smad and NF-κB/p65 signaling.

## DISCUSSION

We have demonstrated that TGF-β signaling is participated in the development of chronic AAN [12]. The present study provided new evidence for a protective role of Smad7, a negative regulator of TGF-β/Smad signaling, in the pathogenesis of chronic AAN. Importantly, we also reported here that Smad7 was an effective therapeutic agent for chronic AAN.

In this study, we found that loss of renal Smad7 may be a key mechanism leading to progression of chronic AAN. This was supported by the finding that genetic deletion of Smad7 enhanced a rapidly progressive renal injury in mice with chronic AAN. In contrast, restored renal Smad7 locally on Smad7 KO mice was capable of preventing the development of chronic AAN. These results demonstrated a protective role for Smad7 in the pathogenesis of chronic AAN, which is consistent with previous studies in a variety of kidney diseases [14-22].

The most important finding in this study is that Smad7 may be a therapeutic agent for chronic AAN. This was supported by the finding that ultrasound-mediated gene transfer of Smad7 into the diseased kidneys with established chronic AAN in Smad7 WT mice at day 14 was capable of attenuating AA-induced progressive renal injury at day 42. These findings were in consistent with previous studies in a number of kidney disease models in which overexpression of Smad7 attenuates renal inflammation and fibrosis in obstructive nephropathy [14], remnant kidney disease [15, 16], autoimmune nephritis [17], diabetic nephropathy [20], and hypertensive nephropathy [21, 22]. However, it should also be pointed out that the therapeutic effect of Smad7 on chronic AAN was based on one time point study at day 42 after Smad7 treatment from day 14 of chronic AA. A prolong therapeutic effect of Smad7 on chronic AAN may be warranted. In addition, outcomes from this study may not be applied directly to the chronic AAN conditions in humans due to the potential difference in inflammatory responses between mice and humans. Nevertheless, the finding from this study provided new evidence for the therapeutic potential of Smad7 in chronic AAN.

There are two major mechanisms by which Smad7 protects against chronic AAN. First, consistent with a negative regulator of Smad7 in TGF-β/Smad signaling [10, 11], Smad7 may act by inhibiting the activation of TGF-β/Smad signaling, thereby blocking renal fibrosis, a major pathological feature of chronic AAN. Indeed, loss of renal Smad7 was found in the kidney with chronic AAN, which may account for TGF-β/Smad3-mediated renal ﬁbrosis. The functional importance of Smad7 in chronic AAN was demonstrated by the findings that deletion of Smad7 aggravated but restoration of Smad7 locally in the kidneys of Smad7 KO mice prevented AA-induced, TGF-β/Smad3-mediated progressive renal fibrosis. In addition, inhibition of TGF-β/Smad3-mediated renal fibrosis by overexpressing renal Smad7 in the established chronic AAN further supported the inhibitory role of Smad7 in progressive chronic AAN and may also well explain the therapeutic effect of Smad7 on chronic AAN. Thus, blockade of TGF-β/Smad3-mediated renal fibrosis could be a mechanism by which Smad7 protects against chronic AAN.

Another mechanism for the protective role of Smad7 in chronic AAN may be due to its inhibitory role in the NF-κB-dependent renal inﬂammation. It is now clear that renal inflammation is also involved in chronic AAN [23, 24]. NF-κB is a critical signaling pathway of the inflammatory cascade. We have previously reported that Smad7 functions as an inhibitor of NF-κB signaling to block NF-κB-dependent renal inflammation *in vitro* and *in vivo* [13, 16-22]. Previous study in an experimental AAN model demonstrated that progression of chronic AAN is associated with infiltration of macrophages and T cells [25]. In line with this ﬁnding, the present study added new evidence that the development of renal inflammation in chronic AAN was associated with a marked activation of NF-κB signaling, which was negatively regulated by Smad7 because deletion of Smad7 enhanced but restored renal Smad7 in Smad7 KO mice or overexpression of Smad7 in the diseased WT mice inhibited NF-κB-mediated renal inflammation including upregulation of TNFα, MCP-1, and infiltration of macrophages and T cells in chronic AAN.

In conclusion, Smad7 plays a protective role in chronic AAN. Inhibition of TGF-β/Smad3 and NF-κB signaling pathways may be mechanisms by which Smad7 attenuates AA-induced renal fibrosis and inflammation. In addition, the ability of overexpression of renal Smad7 to inhibit the established chronic AAN suggests that Smad7 may have therapeutic potential for chronic AAN.

## MATERIALS AND METHODS

### Animal models

A chronic AAN model was induced in genetically identical littermate Smad7 KO and wild-type (WT) mice (CD-1 background mice, male, aged 6–8weeks, 26-31g) by intraperitoneal injection of AA (Sigma, St. Louis, MO) at a dose of 5mg/kg every other day for 6 weeks, as described previously [12]. Smad7 KO mice were generated by functionally deleting exon I in the Smad7 gene as previously described [26]. Groups of 6 mice were killed at 42 days after the initial AA injection for histology, immunohistochemistry, real-time PCR, and western blot analysis. In addition, groups of 6 normal Smad7 KO and WT mice received saline injection were used as normal age-matched control.

To further investigate the role of renal Smad7 in the development of chronic AAN, a rescued study was performed in Smad7 KO mice (male, aged 6-8 weeks, 26-31g) by restoring Smad7 locally into the kidneys of Smad7 KO mice using the ultrasound-microbubble gene transfer technique (see below), which was performed immediately after intraperitoneal injection of AA. Groups of 6 Smad7 KO mice were used. Control mice with chronic AAN had the same procedure but received empty vector treatment. In addition, a group of mice with chronic AAN without ultrasound treatment and a group of normal Smad7 KO mice treated with saline were used as controls.

To test the therapeutic effect of Smad7 in the established chronic AAN, groups of 6 mice with chronic AAN induced in Smad7 WT mice were randomized into three groups at day 14 after the initial AA injection. Group 1 animals received AA without any treatment (AAN); Group 2 mice received AA but treated with empty vector control (VC) from day 14 onwards after the initial injection of AA; Group 3 mice received AA and treated with Smad7 (Smad7) from day 14 onwards after the initial injection of AA. In addition, a groups of AAN mice (n=6) at day 14 before treatment were euthanized as disease control before Smad7 treatment and a group of 6 normal age-matched mice treated with saline were used as normal control. Smad7 gene transfer was performed in groups of 6 Smad7 WT mice with established AAN at day 14 as described below. The experimental procedures were approved by the Institutional Animal Experimentation Ethics Committee (Permit No. 12-352).

### A non-invasive ultrasound-microbubble-mediated Smad7 gene transfer into kidneys

A non-invasive ultrasound-microbubble-mediated Smad7 gene transfer was performed following the previous protocol [17, 21]. Briefly, pTRE_2_-Flag-M2-Smad7 and Tet-on plasmids (100ug/mouse, respectively) were mixed with Sonovue (Bracco Diagnostics, Princeton, NJ, USA) in a 1:1 ratio (volume:volume). The mixture (400ul) was injected into Smad7 KO (for the rescued study) or Smad7 WT (for the treatment study) mice via tail vein, followed by placing the ultrasound probe on the skin of the mouse back opposite to the bilateral kidneys with a plus-wave output (2 W/cm^2^) for a total of 5 min with 30 seconds intervals. After ultrasound treatment, 200ug/ml of doxycycline (Sigma, St, Louis, MO) were injected intraperitoneally, followed by the addition of doxycycline in the daily drinking water (200ug/ml) for the entire study period. Control ultrasound treatment group had the same protocol but received the pTRE_2_-Tet-on empty vectors without Smad7. According to the previous studies that ultrasound-microbubble-mediated Smad7 transgene expression peaked at days 3-7 and declined 2 weeks later [17, 21], Smad7 gene transfer was repeated every 2 weeks until animals were sacrificed.

The experimental procedures were approved by the Institutional Animal Experimentation Ethics Committee (Permit No. 12-352).

### Renal function and proteinuria

Twenty-four hour urine samples were collected in all animals in the metabolic cages at time 0 (before induction of AAN) and at weeks 2,4,6 after induction of AAN for proteinuria assay. Urine protein levels were measured using the Quick start Bradford Dye Reagent (BioRAD). Serum creatinine was used to evaluate renal function. Levels of serum creatinine were detected by the Enzymatic creatinine LiquiColor Reagent (Stanbio Laboratory, Boerne, TX), according to the manufacturer's instructions.

### Histology and immunohistochemistry

Histological injury was performed in 4-μm methyl Carnoy's-ﬁxed parafﬁn sections stained with

Periodic acid-Schiff (PAS). Immnunostaining was performed on parafﬁn sections using a microwave-based antigen retrieval technique [27]. Primary antibodies used in the study were as followed: collagen I (Southern Technology, Birmingham, AL), α-SMA (Sigma, St. Louis, MO), TNFα, MCP-1, TGF-β1, phospho-Smad2/3 (Santa Cruz Biotechnology, Santa Cruz, CA), phospho-NFκB/p65, CD3 (Abcam, Cambridge, MA), and F4/80 (Serotec, Oxford, UK). After being immunostained with the secondary antibodies, sections were developed with diaminobenzidine to produce a brown color. All slides were counterstained with hematoxylin except for phospho-Smad2/3 and phospho-NFκB/p65 immunodetection. The percentage of positive staining for collagen I, TNFα, MCP-1, TGF-β1 was measured by using a quantitative image-analysis system (Image-Pro Plus 6.5, Media Cybernetics, Silver Spring, MD) [28, 29], while the number of positive phopsho-p65, phospho-Smad2/3, CD3, F4/80+ cells in the tubulointerstitium were counted under high-power fields (×40) by means of a 0.0625-mm^2^ graticule fitted in the eyepiece of the microscope and expressed as cells per millimeters squared.

### Real-time PCR

RNA was collected from renal tissues and puriﬁed by an RNeasy kit according to the manufacturer's instructions (Qiagen, Valencia, CA), and real-time PCR was performed with Sybergreen on an Opticon real-time PCR machine (MJ Research, Waltham, MA) as previously described. [28-30] Primers used for detection of mRNA expression of collagen I, α-SMA, TGF-β1, MCP-1, TNFα, and GAPDH were described previously [28-30]. House keep gene GAPDH was used as an internal standard. The ratio for the mRNA was examined against GAPDH and was expressed as mean±SE.

### Western blot analysis

Protein from renal tissues were extracted with RIPA lysis buffer and analyzed by Western blotting as previously described [28-30]. Brieﬂy, after protein was transferred onto a nitrocellulose membrane, the membrane was incubated at 4°C overnight with primary antibodies against phospho-p65 (ser276), phospho-IκBα (ser32) and IκBα (Cell Signaling), p65, phospho-Smad2/3, Smad7 and Smad2/3 (Santa Cruz), collagen I (Southern Biotech), α-SMA (Sigma), GAPDH (Chemicon, Temecula, CA), followed by the LI-COR IRDye 800-labeled secondary antibodies (Rock-land Immunochemicals, Gilbertsville, PA). The signals were detected with an Odyssey Infrared Imaging System (Li-COR Biosciences, Lincoln, NE, USA) and quantified with Image J (National Institutes of Health, Bethesda, MD, USA). The ratio for the protein examined was normalized against GAPDH.

### Statistical analysis

Data obtained from this study are expressed as means±SE. Statistical analyses were performed using one-way analysis of variance followed by a Newman-Keuls posttest (Prism 5.0 GraphPad Software, San Diego, CA).

## SUPPLEMENTARY MATERIAL, FIGURES


