# Supplementary material for: Smad7 protects against chronic aristolochic acid nephropathy in mice
Source: Oncotarget. 2015 Mar 30;6(14):11930–44. doi: 10.18632/oncotarget.3718 (PMC4494914; doi:10.18632/oncotarget.3718)
Supplement: Supplementary file 1 [file oncotarget-06-11930-s001.pdf]

Smad7 protects against chronic aristolochic acid nephropathy in mice

Supplementary Material

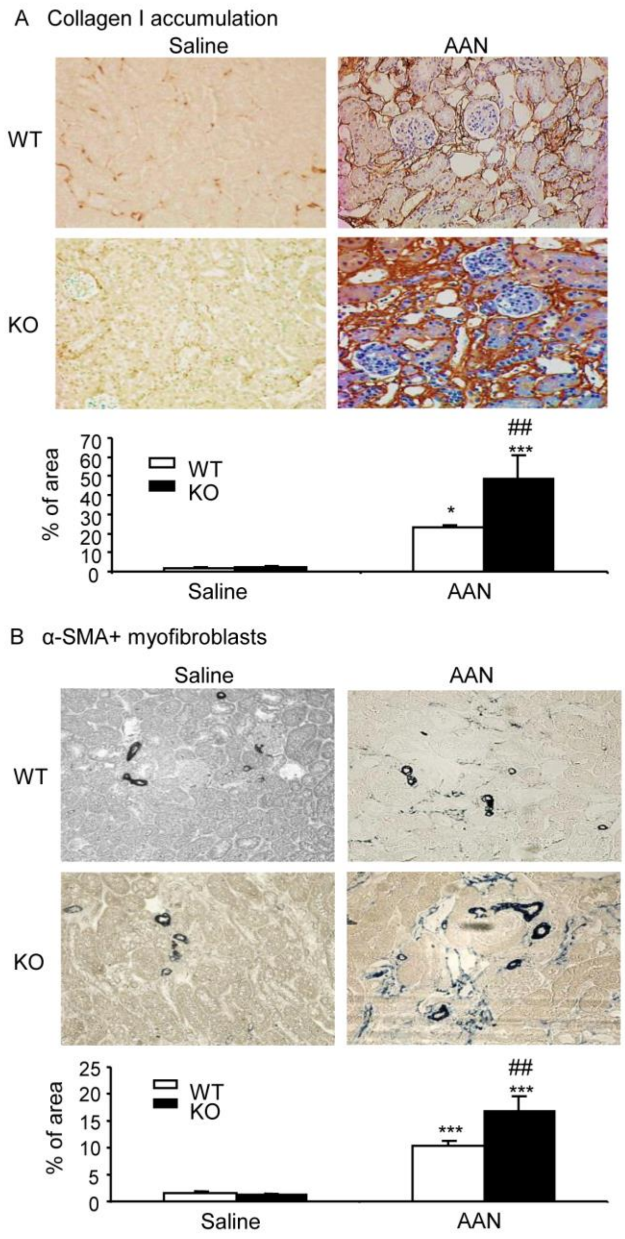

Figure S1. Immunohistochemistry detects that deletion of Smad7 enhances renal collagen I deposition and α-SMA+ myofibroblast accumulation in chronic AAN at day 42. A: Collagen I deposition. **B:** α-SMA+ myofibroblasts. Results show that compared with the WT mice, Smad7 KO mice show a significant increase in both fibrotic markers in the kidney with chronic AAN at day 42. Data are expressed as mean ± SE for groups of 6 mice. $^{*}P<0.05$, $^{**}P<0.01$, $^{***}P<0.001$ compared with the saline control mice. $^{\#}P<0.05$, $^{\#\#}P<0.01$, $^{\#\#\#}P<0.001$ compared with Smad7 WT mice with chronic AAN mice. Magnification: x200.

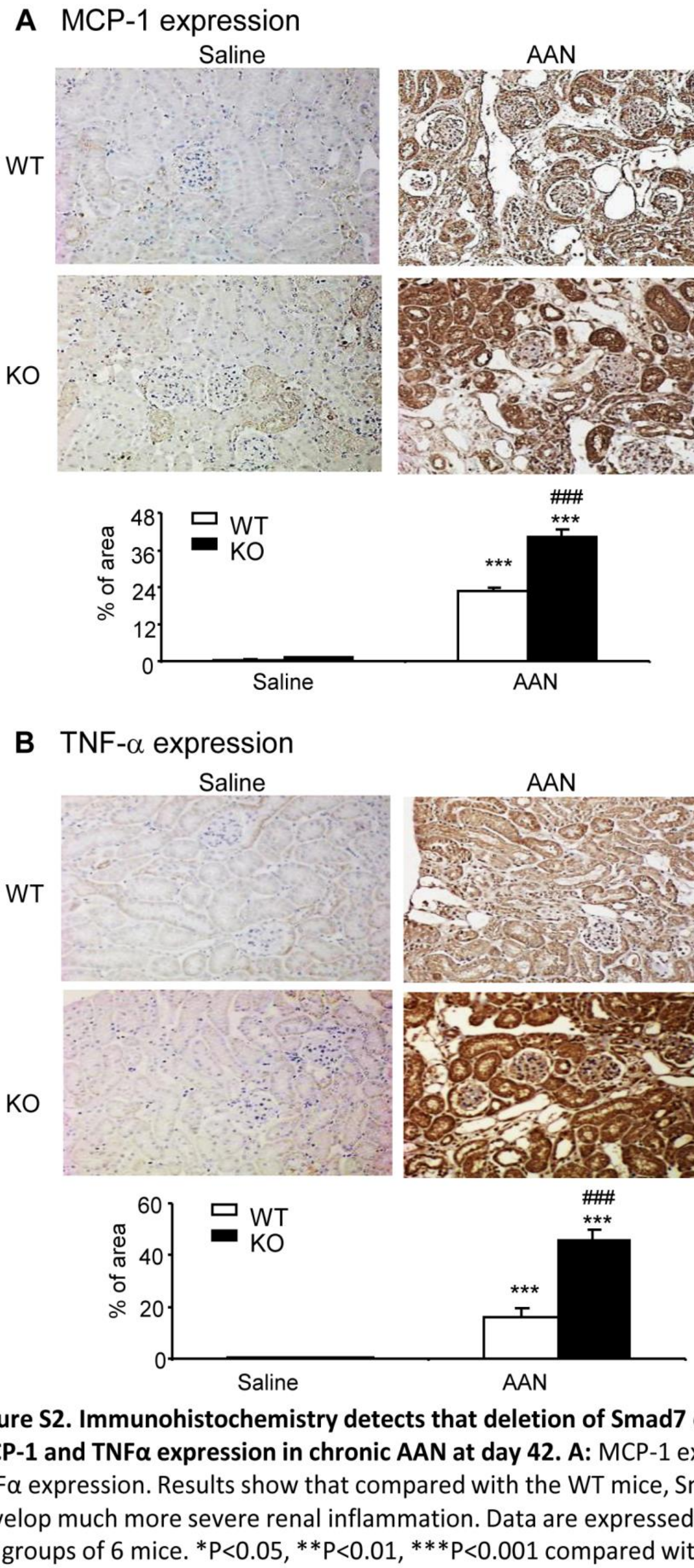

Figure S2. Immunohistochemistry detects that deletion of Smad7 enhances renal MCP-1 and TNFα expression in chronic AAN at day 42. A: MCP-1 expression. **B:** TNFα expression. Results show that compared with the WT mice, Smad7 KO mice develop much more severe renal inflammation. Data are expressed as mean ± SE for groups of 6 mice. *P<0.05, **P<0.01, ***P<0.001 compared with the saline control mice. $^{\#}$P<0.05, $^{\#\#}$P<0.01, $^{\#\#\#}$P<0.001 compared with Smad7 WT mice with chronic AAN mice. Magnification: x200.

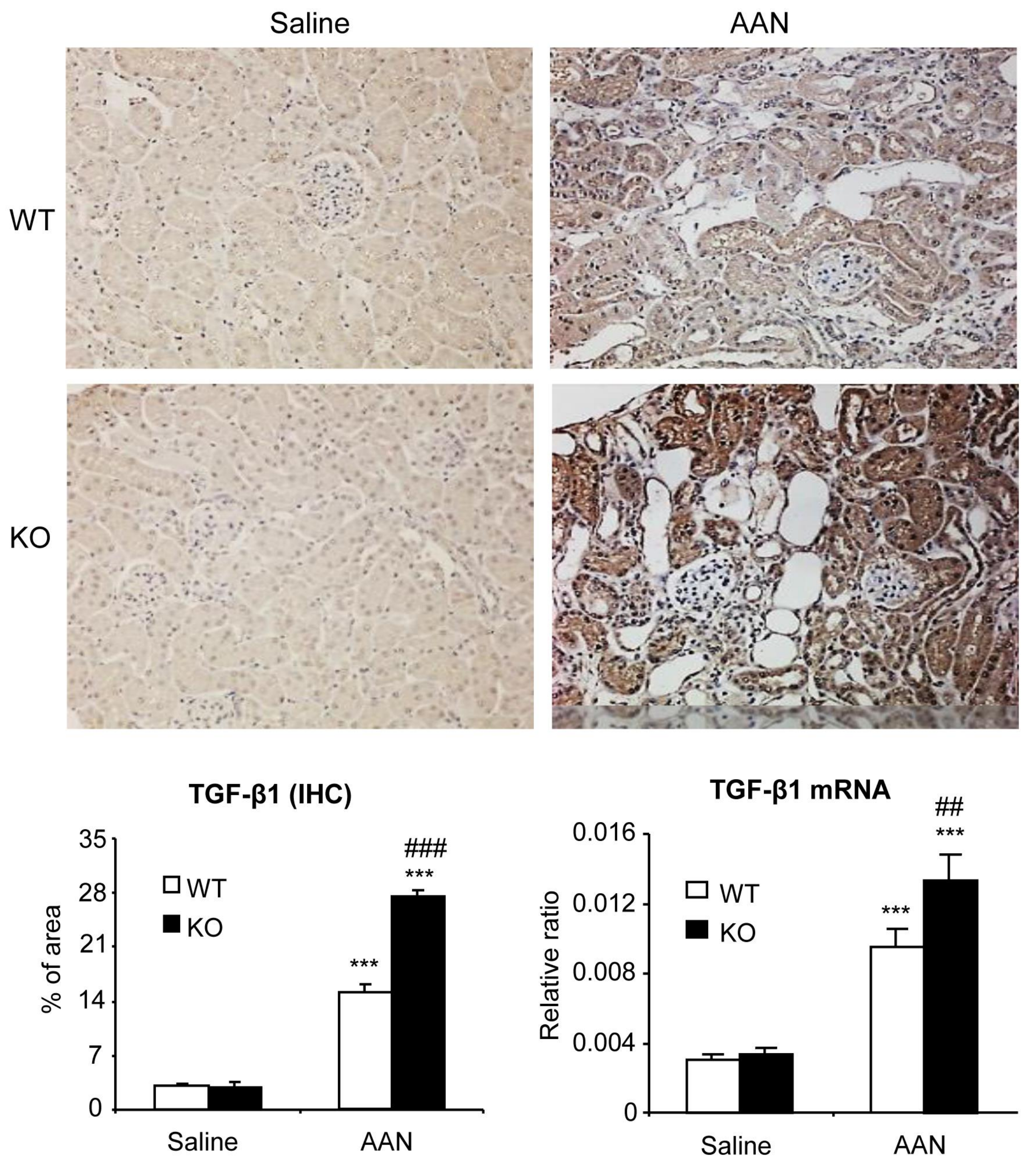

Figure S3. Real-time PCR and immunohistochemistry detect that deletion of Smad7 enhances renal TGFβ1 expression in chronic AAN at day 42. Note that compared to normal Smad7 WT mice, TGF-β1 mRNA and protein is significantly upregulated in the AAN kidney of Smad7 KO mice. Data are expressed as mean $\pm$ SE for groups of 6 mice. *P<0.05, **P<0.01, ***P<0.001 compared with saline control mice. $^{\#}$P<0.05, $^{\#\#}$P<0.01, $^{\#\#\#}$P<0.001 compared with Smad7 WT mice with chronic AAN mice. Magnification: $\times$200.

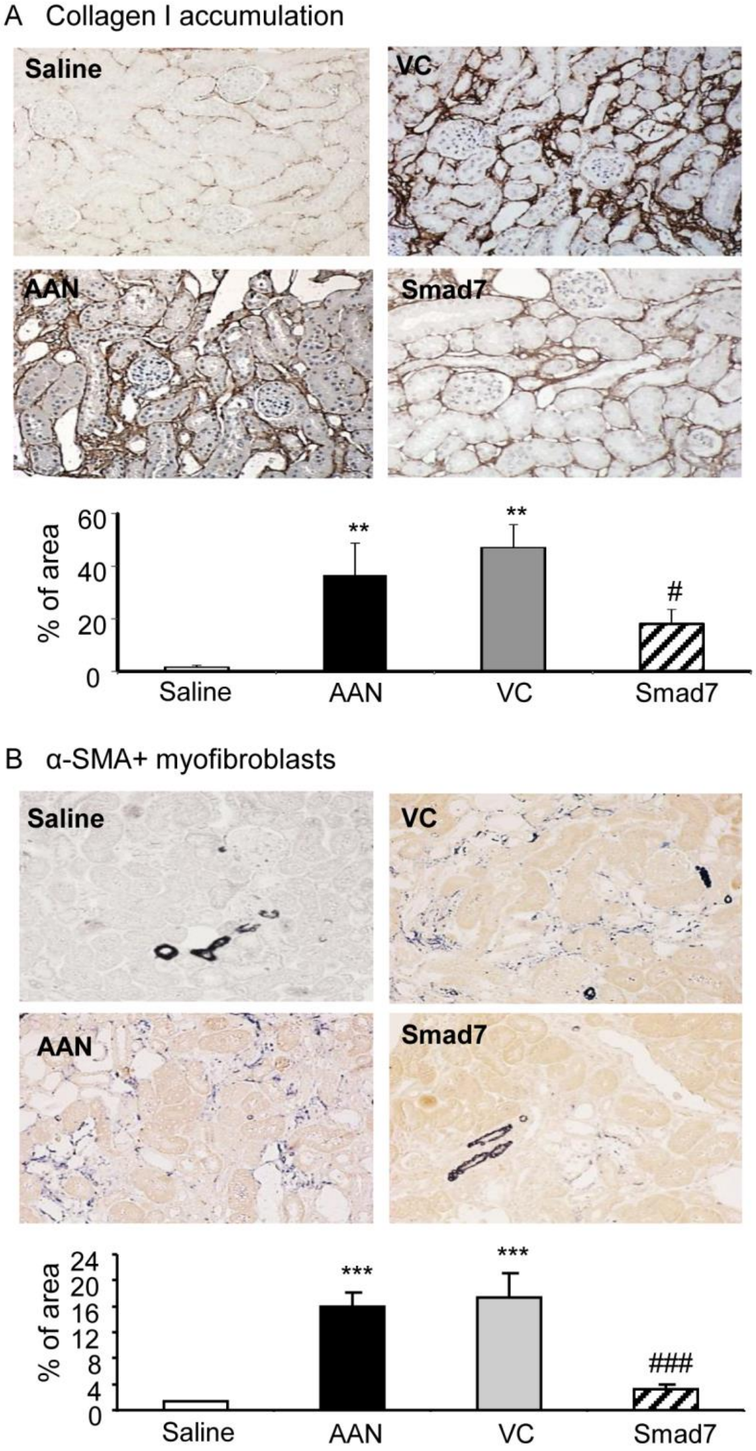

Figure S4. Immunohistochemistry reveals that restored renal Smad7 attenuates AA-induced renal fibrosis in Smad7 KO mice at day 42. A: Collagen I expression. **B:** α-SMA+ myofibroblast accumulation. Results show that compared to Smad7 KO mice with chronic AAN treated with or without vector control (VC), restored renal Smad7 on Smad7 KO mice largely inhibits renal fibrosis. Data are expressed as mean $\pm$ SE for groups of 6 mice. *P<0.05, **P<0.01, ***P<0.001 compared with the saline control mice. $^{\#}$P<0.05, $^{\#\#}$P<0.01, $^{\#\#\#}$P<0.001 compared with Smad7 KO mice with chronic AAN treated with or without VC. Magnification: x200.

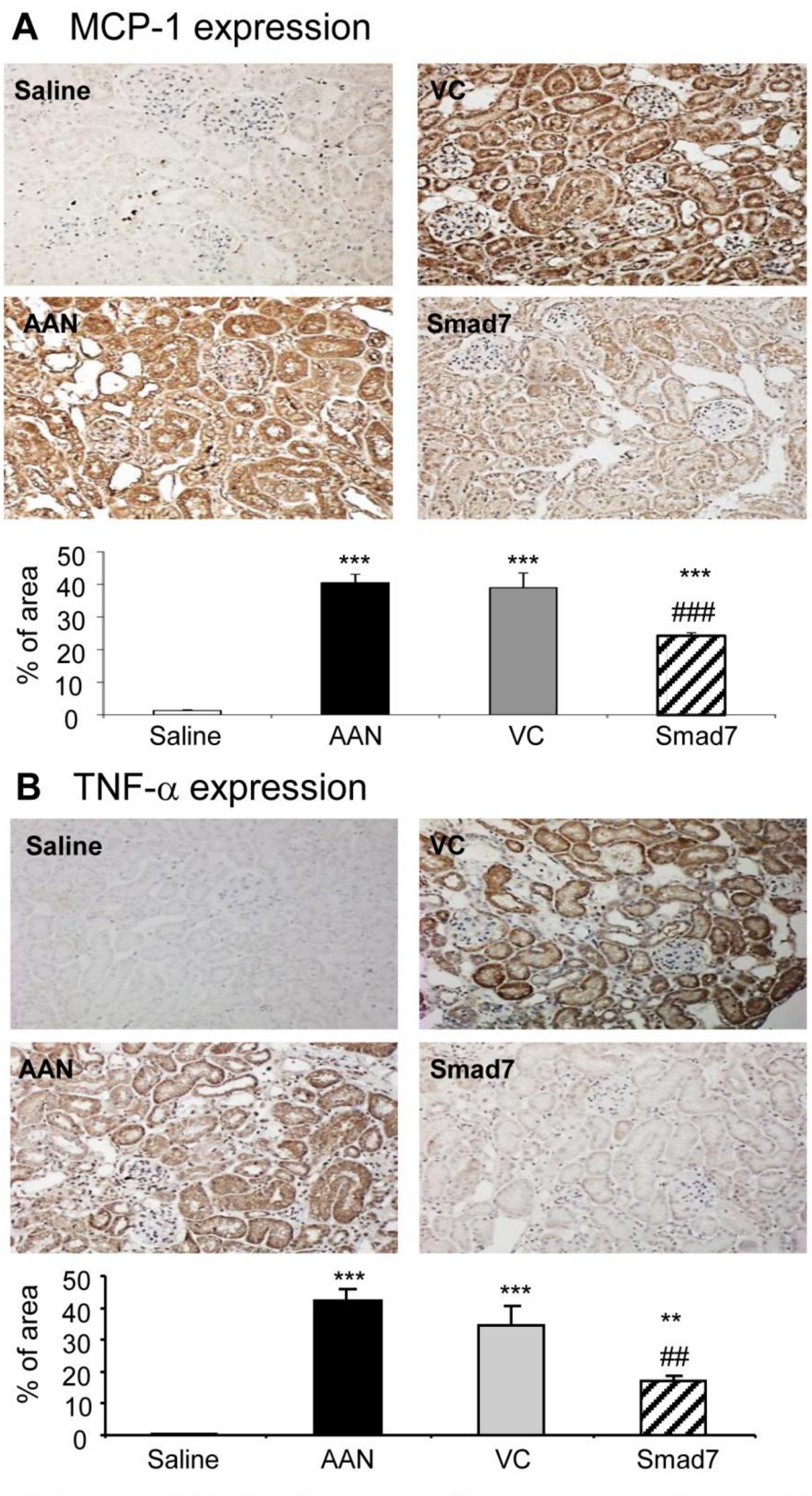

Figure S5. Immunohistochemistry reveals that restored renal Smad7 attenuates AA-induced renal inflammation in Smad7 KO mice at day 42. A: MCP-1 expression. **B:** TNFα expression. Results show that compared to Smad7 KO mice with chronic AAN treated with or without vector control (VC), restored renal Smad7 on Smad7 KO mice largely inhibits renal inflammation. Data are expressed as mean ± SE for groups of 6 mice. *P<0.05, **P<0.01, ***P<0.001 compared with the saline control mice. #P<0.05, ##P<0.01, ###P<0.001 compared with Smad7 KO mice with chronic AAN treated with or without VC. Magnification: x200.

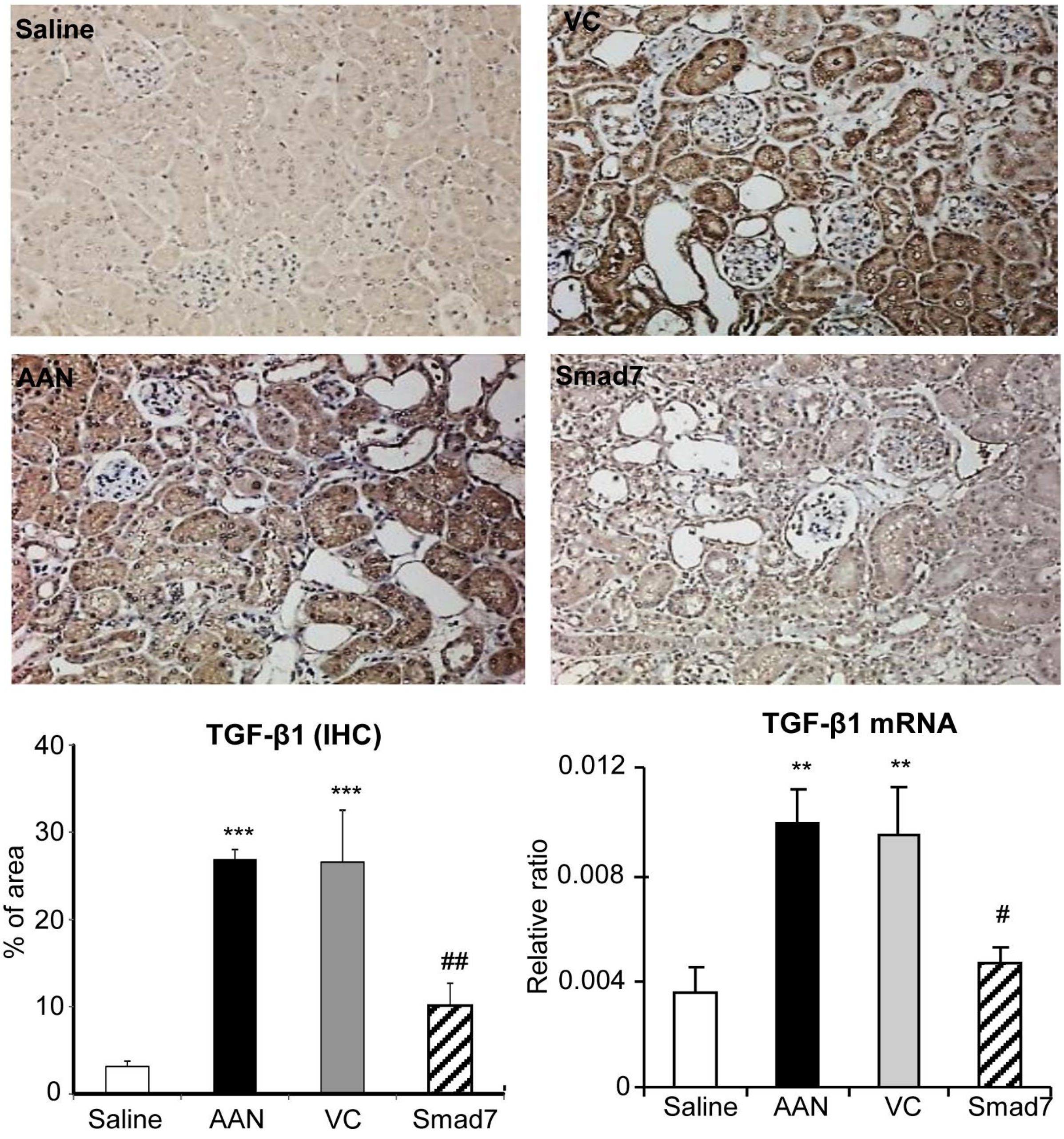

Figure S6. Real-time PCR and immunohistochemistry detect that restored renal Smad7 inhibits TGF-β1 expression in the kidney of Smad7 KO mice with chronic AAN. Note that compared to Smad7 KO mice with chronic AAN treated with or without vector control (VC), restored renal Smad7 largely blocks expression of TGF-β1 in the AAN kidney of Smad7 KO mice. Data are expressed as mean $\pm$ SE for groups of 6 mice. *P<0.05, **P<0.01, ***P<0.001 compared with saline control mice. $^{\#}$P<0.05, $^{\#\#}$P<0.01, $^{\#\#\#}$P<0.001 compared with Smad7 KO mice with chronic AAN treated with or without VC. Magnification: x200.

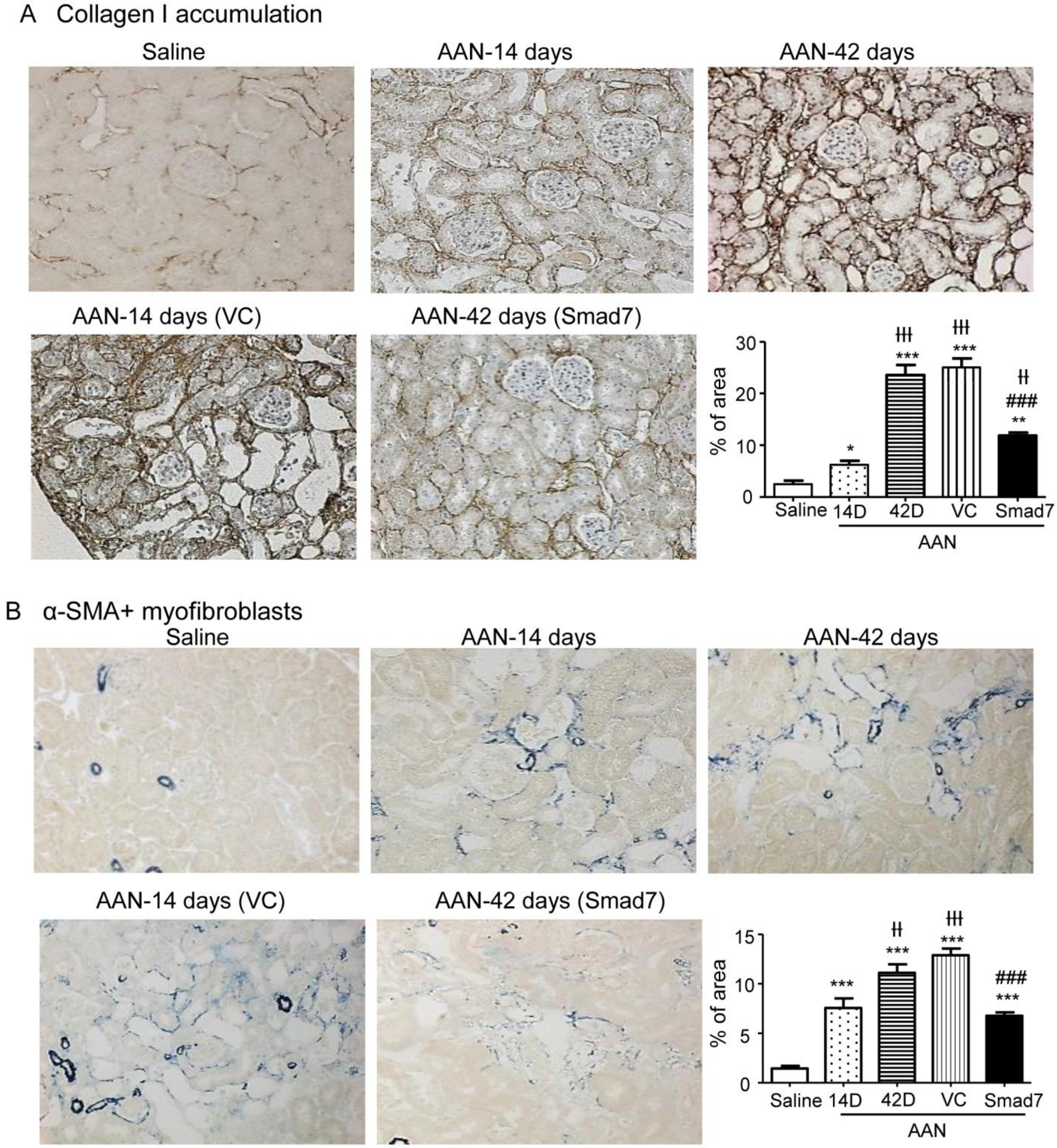

Figure S7. Immunohistochemistry shows that overexpression of Smad7 in the kidney attenuates renal fibrosis in Smad7 WT mice with established chronic AAN. A: Collagen I expression. **B:** α-SMA+ myofibroblasts. Results show that compared to Smad7 WT mice with chronic AAN treated with or without vector control (VC), Smad7 treatment locally in the kidney with established AAN at day 14 blocks renal fibrosis at day 42. Data are expressed as mean ± SE for groups of 6 mice. *P<0.05, *P<0.01, ***P<0.001 compared with saline control mice. $^{\#\#\#}$p<0.001 compared with Smad7 WT mice with chronic AAN treated with or without VC. †P<0.05, ††P<0.01, †††P<0.001 compared with day 14 disease before Smad7 treatment. Magnification: x200.

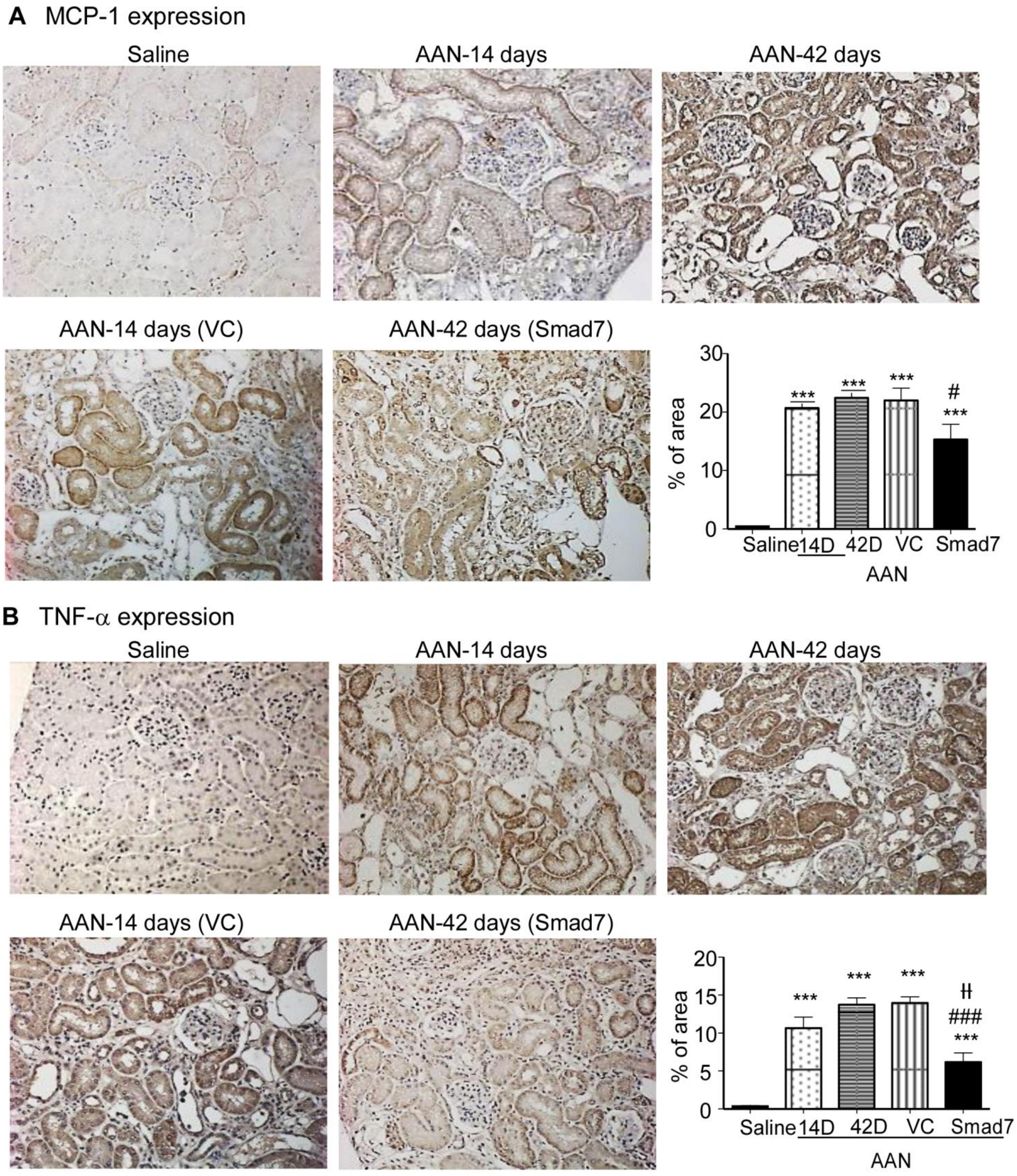

Figure S8. Immunohistochemistry shows that Smad7 therapy attenuates renal inflammation in Smad7 WT mice with established chronic AAN. A: MCP-1 expression. **B:** TNFα expression. Results show that compared to Smad7 WT mice with chronic AAN treated with or without vector control (VC), Smad7 treatment locally in the kidney with established AAN at day 14 blocks renal MCP-1 and TNFα expression at day 42. Data are expressed as mean ± SE for groups of 6 mice. ***P<0.001 compared with saline control mice. $^{\#}P<0.05$, $^{\#\#\#}p<0.001$ compared with Smad7 WT mice with chronic AAN treated with or without VC. $^{\dagger\dagger}P<0.01$ compared with day 14 disease before Smad7 treatment. Magnification: x200.

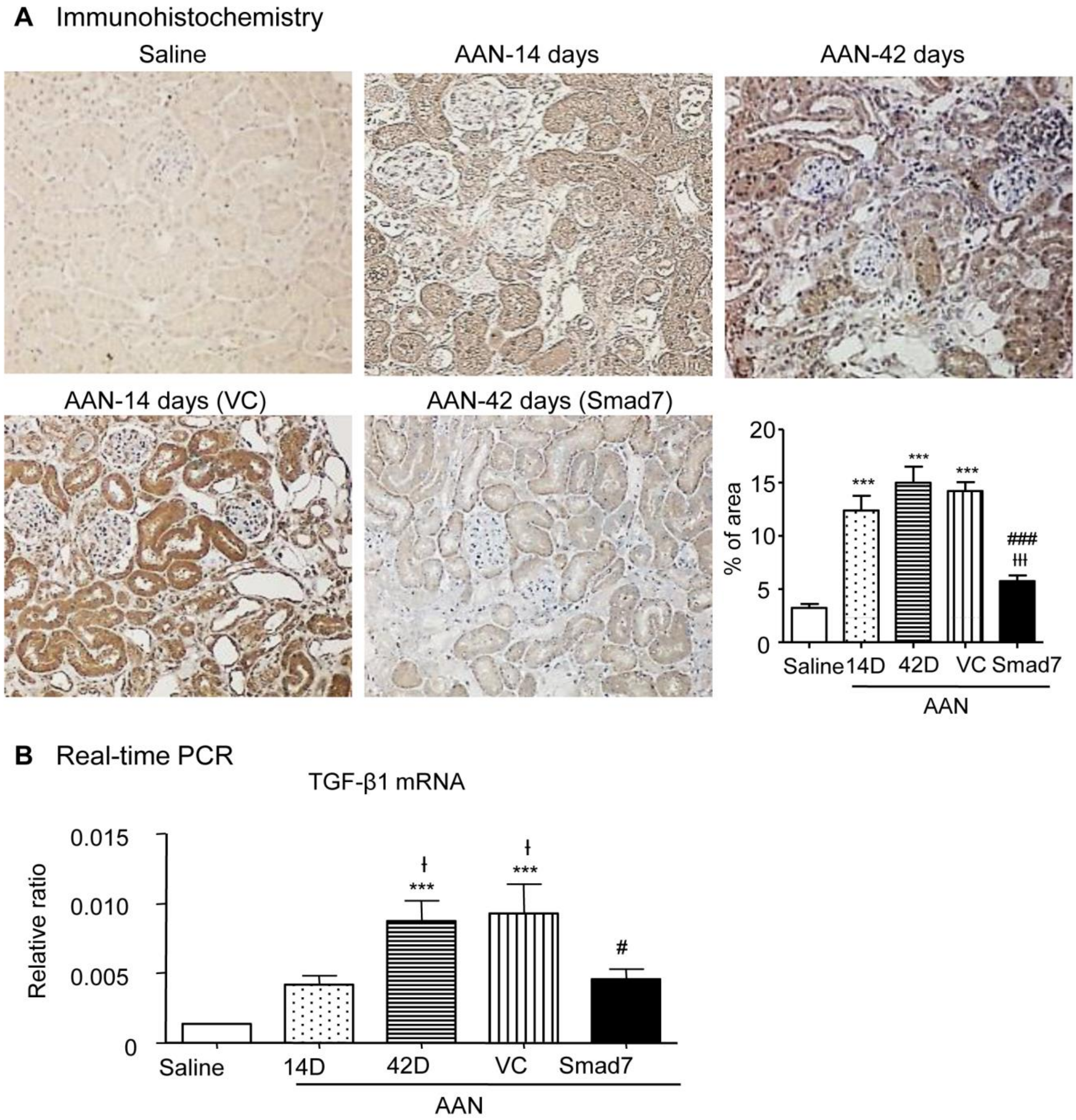

Figure S9. Smad7 treatment locally in the kidney with established chronic AAN blocks expression of TGF-β1. A: Immunohistochemistry. **B**: real-time PCR. Note that compared to Smad7 WT mice with chronic AAN treated with or without vector control (VC), Smad7 treatment locally in the kidney with established AAN at day 14 largely blocks a marked activation of TGF-β1 in the AAN kidney at day 42. Data are expressed as mean ± SE for groups of 6 mice. *P<0.05, **P<0.01, ***P<0.001 compared with saline control mice. $^{\#}$P<0.05, $^{\#\#}$P<0.01, $^{\#\#\#}$P<0.001 compared with Smad7 WT mice with chronic AAN at day 42 treated with or without VC. †P<0.05, †††P<0.001 compared with day 14 disease before Smad7 treatment. Magnification: x200.
